# Self-Powered Au/ReS_2_ Polarization Photodetector with Multi-Channel Summation and Polarization-Domain Convolutional Processing

**DOI:** 10.3390/s25175375

**Published:** 2025-09-01

**Authors:** Ruoxuan Sun, Guowei Li, Zhibo Liu

**Affiliations:** 1 The Key Laboratory of Weak Light Nonlinear Photonics, Ministry of Education, School of Physics, Teda Applied Physics Institute, State Key Laboratory of Photovoltaic Materials and Cells, Nankai University, Tianjin 300071, China; 9820240064@nankai.edu.cn (R.S.); 1120230126@mail.nankai.edu.cn (G.L.); 2 The Collaborative Innovation Center of Extreme Optics, Shanxi University, Taiyuan 030006, China

**Keywords:** ReS_2_, self-powered photodetector, polarization imaging, computational sensing

## Abstract

Polarization information is essential for material identification, stress mapping, biological imaging, and robust vision under strong illumination, yet conventional approaches rely on external polarization optics and active biasing, which are bulky, alignment-sensitive, and power-hungry. A more desirable route is to encode polarization at the pixel level and read it out at zero bias, enabling compact, low-noise, and polarization imaging. Low-symmetry layered semiconductors provide persistent in-plane anisotropy as a materials basis for polarization selectivity. Here, we construct an eight-terminal radial ‘star-shaped’ Au/ReS_2_ metal-semiconductor junction array pixel that operates in a genuine photovoltaic mode under zero external bias based on the photothermoelectric effect. Based on this, electrical summation of phase-matched multi-junction channels increases the signal amplitude approximately linearly without sacrificing the two-lobed modulation depth, achieving ‘gain by stacking’ without external amplification. The device exhibits millisecond-scale transient response and robust cycling stability and, as a minimal pixel unit, realizes polarization-resolved imaging and pattern recognition. Treating linear combinations of channels as operators in the polarization domain, these results provide a general pixel-level foundation for compact, zero-bias, and scalable polarization cameras and on-pixel computational sensing.

## 1. Introduction

Determining the polarization state of light enables functions that intensity-only imagers cannot deliver: material discrimination, stress mapping, tissue contrast, and robust vision under glare [1,2,3,4]. Conventional approaches rely on external polarizers and analyzers aligned in front of photodiodes. These assemblies are accurate but bulky, alignment-sensitive, and power-hungry when active modulation is involved. A more compact route is to let the detector itself encode polarization and to read it in a photovoltaic regime, so that the scene’s polarization is converted to current directly at the device plane without auxiliary optics or electrical bias. Low-symmetry layered semiconductors are natural candidates for such in-detector polarimetry [3,5,6,7,8,9]. In rhenium disulfide (ReS_2_), a distorted 1T lattice establishes persistent in-plane anisotropy, with optical matrix elements and carrier transport both depending on crystal direction [2,8,10,11,12,13,14]. Devices exploiting this anisotropy have reported polarization contrast, yet most operate in photoconductive or photogating modes under applied bias [2,11,15]. Bias improves gain but brings dark current, noise, and Joule heating, which complicate calibration and obscure weak signals. By contrast, devices relying on localized photovoltaic (driven by built-in electric field) or photothermoelectric (PTE) effects (driven by temperature difference) can separate photocarriers in the absence of bias, resulting in clean short-circuit currents and open-circuit voltages set solely by the illumination [16,17,18,19,20,21]. The challenge is architectural: to preserve polarization contrast, raise absolute signal level, and scale naturally to imaging—all while remaining zero-bias.

The offloading part of the computation to the sensor can significantly reduce redundant data and minimize data transfer between the sensor and the processor [12,22]. As a result, real-time image processing at the sensor level has become an important direction in photodetector development. On-chip convolution for feature extraction represents one of the most essential preprocessing functions, which typically requires tunable photoresponsivity. Conventionally, responsivity modulation is achieved by adjusting the gate voltage [23,24].

Here we introduce a self-powered polarization photodetector based on an eight-terminal Au/ReS_2_ junction array patterned in a radial ‘star’ layout. Each metal finger forms its own junction to a common ReS_2_ flake. For a metal–semiconductor–metal (MSM) device, the two metal–semiconductor junctions are symmetric. When uniform illumination is applied to the entire device, no self-powered photocurrent can be generated. This is because even if Schottky built-in fields or PTE effects exist at the metal–semiconductor interfaces, the photocurrents induced at the two symmetric junctions cancel each other out, resulting in no net self-powered photoresponse [11,16]. Therefore, only when the two junctions operate independently can the internal fields or temperature gradients be effectively modulated by light to yield a self-powered photocurrent. Ying et al. demonstrated this concept in a Au/Ti–SnTe–Ti/Au photodetector with two symmetric Schottky junctions, where the photovoltage could be tuned by moving the light spot between the junctions, enabling a bipolar response [16]. In a similar manner, our eight-terminal Au/ReS_2_ device contains four pairs of symmetric contacts. To investigate the mechanism by which the Au/ReS_2_ metal–semiconductor junction drives zero-bias photocurrent and to fully exploit its self-powered functionality, we selectively illuminated specific contact regions. Under illumination, each junction produced a self-powered signal whose magnitude depended on the angle between the incident linear polarization and the crystalline axes of ReS_2_. The eight spokes act as an angular sampler distributed over 360°, providing complementary channels: spokes close to the high-absorption axis respond strongly, whereas orthogonal spokes serve as references. Because the junctions are individually addressable, their outputs can be electrically combined. Summing non-collinear spokes yields a proportional increase in current—an intrinsic signal build-up without external gain—without diluting the anisotropic contrast, which remains set by the material. Readout at zero external bias reduces drift and simplifies packaging for dense tiling. The *I*–*V* curves show nonzero short-circuit current and open-circuit voltage at zero bias, confirming that the built-in electric or temperature field of the Au/ReS_2_ metal–semiconductor interface, rather than external bias-assisted photoconduction, drives the response.

We further show that the same pixel architecture supports polarization-resolved imaging and pattern recognition. In this work, we exploit the polarization-sensitive photoresponse of the photodetector to perform image convolution. Unlike gate voltage control, this approach eliminates the need for electrode and gate dielectric design, thereby simplifying device fabrication. Projecting polarization-encoded test patterns onto the active region and mapping the eight-channel current set allows the reconstruction of polarization-dependent features—demonstrated here with alphanumeric targets. The transient response lies in the millisecond range, limited primarily by junction capacitance and series/interconnect resistance rather than slow trapping, and the output remains stable over repeated cycling. Importantly, the star pixel is lithography-friendly and readily tiled into two-dimensional arrays. Junction grouping (sum, difference, or independent read) can be selected electronically to trade between sensitivity and angular resolution without altering the optics. This study achieves linear polarization detection in ReS_2_ under a zero-bias photovoltaic regime, eliminating bias-induced dark current and drift and establishing a stable, quantifiable baseline. Building on this, we introduce an eight-terminal radial multi-junction architecture that maps the material’s intrinsic anisotropy into angular selectivity and achieves channel-count-scalable signal amplitude via the electrical superposition of independent Schottky paths. Finally, we validate polarization imaging and pattern recognition within the same minimal pixel, outlining a clear engineering route toward compact, zero-bias polarization cameras.

## 2. Results and Discussion

**Figure 1:**
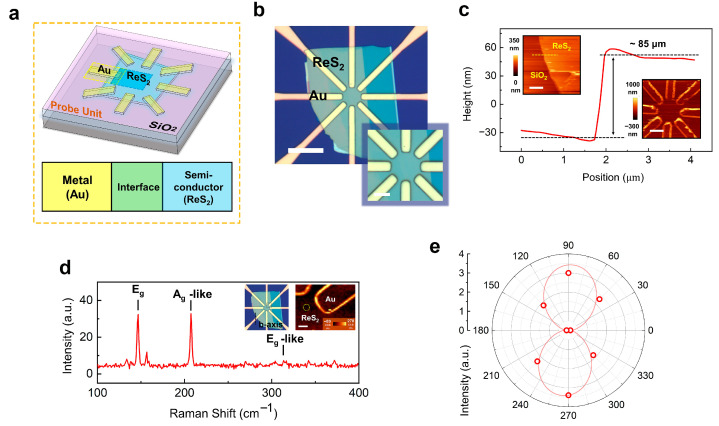
Device structure and axis alignment of the Au/ReS_2_ star photodetector. (**a**) Schematic of the eight−spoke Au/ReS_2_ junction ‘star’ pixel. (**b**) Optical micrograph showing the transferred ReS_2_ bridging all spokes. Scale, 30 μm. Inset scale, 6 μm. (**c**) AFM height and topography images of the device. Left inset scale, 2 μm. Right inset scale, 8 μm. (**d**) Raman spectrum with characteristic modes (Eg ≈ 150 cm^−1^, Ag-like ≈ 210 cm^−1^, Eg-like ≈ 305 cm^−1^); inset, spatial intensity map of the Ag-like peak over the active region. Inset scale, 2 μm. (**e**) Angle-resolved Raman shows a 180° two-lobe pattern; the Re-chain axis is ~3° from the vertical electrode axis, defining the angular reference for polarization measurements and confirming calibrated in-plane anisotropy. The b-axis (Re-chain) crystal orientation based on the Raman results is marked in the inset of Figure 1d.

Candidate ReS_2_ flakes (Xianfeng Nanomaterials Technology Co., Ltd., Nanjing, China) with suitable thickness and lateral dimensions were first transferred onto a SiO_2_/Si substrate using a dry-transfer technique. Subsequently, the device was fabricated by photolithographically defining eight radially arranged gold spokes in a star-shaped configuration (Figure 1a,b). Each spoke forms an individual metal–semiconductor junction, enabling single-junction readout and later electrical grouping (see Supporting Information Section S1 for details). Atomic force microscopy (AFM) height maps resolve the thickness and surface morphology at the Au/ReS_2_ junction (Figure 1c). The ReS_2_ flake is ≈85 nm thick, while the Au electrode measures ≈120 nm at the edge and ≈30 nm at the center; this edge–center difference arises from the gold-removal step during fabrication. The flake is uniform, with no discernible wrinkles or debris. Raman spectroscopy confirms crystalline quality and uniformity (Figure 1d). The spectra exhibit the characteristic E_g_ (~150 cm^−1^), A_g_-like (~210 cm^−1^) and E_g_-like (~305 cm^−1^) modes [25,26,27]. The A_g_-like intensity map (inset) is spatially homogeneous across the junction area, indicating negligible residue or strain gradients. Angle-resolved Raman (Figure 1e) displays a clear two-lobe pattern with 180° periodicity. The intensity maxima identify the Re-chain (b-axis) direction and, when compared with the vertical electrode axis, reveal a small misalignment of ~3°. This value is within our lithography/transfer tolerance and close to the optical stage’s angular resolution, so we regard the alignment as effectively correct for device operation.

The ~3° offset is nevertheless instructive for interpreting the polarization data. It introduces a constant phase shift in the polar photocurrent *I*(*θ*), but does not affect the extracted dichroic ratio or the photovoltaic nature of the response. Accordingly, all fits in subsequent sections include an offset angle *φ* to account for any residual rotation; *φ* agrees with the Raman-derived ~3°, validating our axis registration. Practically, this small deviation has no measurable impact on the multi-junction summation strategy because spokes are separated by 45°, far exceeding the misalignment. For future array scaling, the Raman-assisted registration used here provides a straightforward route to sub-5° alignment between crystal axes and electrode templates, ensuring reproducible angular sampling and minimizing systematic error in polarization imaging.

**Figure 2:**
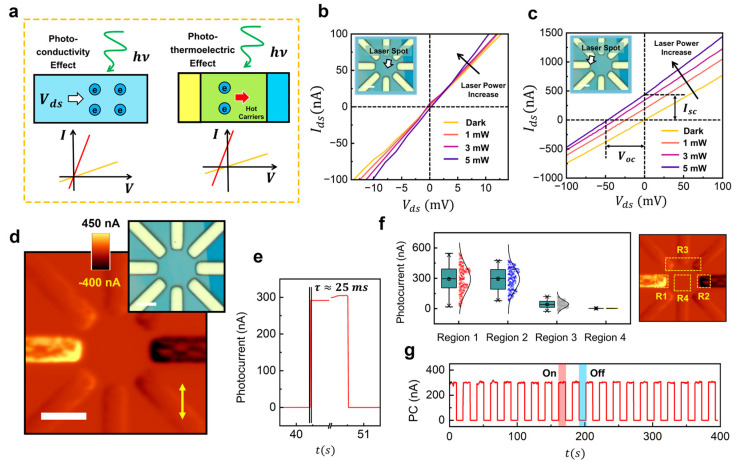
Self-powered operation and locality of the Au/ReS_2_ photodetector. (**a**) Schematic comparison of photoconductive (bias-dependent) and self-powered modes. (**b**) *I*–*V* curves when the laser illuminates the ReS_2_ interior: the slope increases with power but the curve crosses the origin, consistent with photoconductivity. (**c**) *I*–*V* curves with the laser on a Au/ReS_2_ interface: nonzero *I_sc_* at *V_ds_* = 0 and *V_oc_* at *I* = 0 grow with power, demonstrating a field-driven self-powered response (polarity set by the illuminated contact). (**d**) Zero-bias *I_sc_* map from scanning, showing signals confined to metal–semiconductor edges. Scale, 10 μm. Test wavelength, 532 nm. Inset scale, 6 μm (**e**) Transient response with τ=25 ms. (**f**) Photocurrent statistics for four regions (R1–R4); R1/R2 dominate (R2 plotted as abs(I)), whereas a non-wired pad (R3) and bare ReS_2_ (R4) are near noise, indicating low crosstalk. (**g**) Long-term cycling with stable on/off plateaus. Test wavelength, 650 nm.

We next introduce two operating modes relevant to ReS_2_ photodetectors (Figure 2a) and examine the self-powering mechanism of our devices. In the photoconductive mode, illumination increases the channel conductance, and a source–drain bias is required to drive the current. In this case, the *I*–*V* curve remains centered at the origin, with its slope increasing under illumination. In contrast, in the self-powered mode, the built-in field at the metal–semiconductor junction separates photocarriers without the need for an external bias, giving rise to a finite short-circuit current (*I_sc_*) and open-circuit voltage (*V_oc_*). These distinctions are verified by position-dependent *I*–*V* measurements. When the laser is focused at the center of the ReS_2_ flake, far from the contacts, illumination only steepens the *I*–*V* slope without producing an offset—consistent with a purely photoconductive response of the ReS_2_ channel (Figure 2b). In fact, a small focused spot at the channel center yields the same effect as uniform illumination over the entire device, where an external bias is necessary to collect photocurrent [11,16]. By contrast, illuminating the Au/ReS_2_ interface (Figure 2c) generates a distinct self-powered response: nonzero *I_sc_* and *V_oc_* that scale with incident power. These measurements are performed between a pair of opposing electrodes across the flake, so the sign of *I_sc_* directly reflects the polarity of the illuminated junction.

The symmetrical MSM Schottky device is equivalent to two back-to-back Schottky diodes with a channel resistor *R* in series. The total current can be written as

(1) $$\;\;I\left(V\right)=I_{MSM}+\frac{V}{R}\;\;\;$$

where the *I_MSM_* is a nonlinear current determined by two Schottky junctions, ideally exhibiting rectifying or exponential behavior with a characteristic “bend” at a certain bias voltage. In contrast, the *V/R* relationship is strictly linear. If *R* is large, the *I*–*V* curve is expected to bend at relatively high bias. If *R* is small, the bending occurs at lower bias, such that within the measurement voltage window the curve may appear nearly as a straight line through the origin—the intrinsic rectification being effectively masked by the resistance. In both the wide-range *I*–*V* curves (Figure S2) and the narrow-range *I*–*V* curves (Figure 2b,c), no bending is observed, indicating that the Au/ReS_2_ interface forms an ohmic contact with negligible Schottky barrier rectification. The resulting *I*–*V* characteristics are fully consistent with those of a PTE device. Specifically, in the dark state, the device exhibits ordinary resistive behavior—linear and passing through the origin—because the overall transport is governed by ohmic conduction, yielding an essentially straight *I*–*V* line. When the laser is focused at the metal–semiconductor interface, the local temperature rises, generating a temperature gradient Δ*T*. Owing to the difference in Seebeck coefficients Δ*S* between the metal and semiconductor (or between the two electrodes), a thermoelectric voltage is produced:

(2) $$\;\;\;V_{PTE}=∆S\cdot ∆T\;\;$$

Accordingly, the measured *I*–*V* relation can be expressed as

(3) $$I\left(V\right)=\frac{V}{R}+\frac{V_{PTE}}{R}\;\;$$

Under illumination, the *I*–*V* curves exhibit a characteristic lateral shift, with the zero-bias photocurrent increasing approximately linearly with optical power. Moreover, the PTE effect has already been experimentally demonstrated in ReS_2_ and its heterostructures, and due to the strong in-plane anisotropy of ReS_2_, the magnitude of the PTE response shows a pronounced directional dependence [28,29,30].

To verify that the active region is localized at the junctions, we performed scanning-laser photocurrent mapping at zero bias (Figure 2d). The short-circuit current map shows bright lobes confined to Au/ReS_2_ interfaces, with negligible signal over the interior crystal, directly linking the response to the PTE effect. The statistical summary in Figure 2f further quantifies this localization across four representative regions: the two biased contacts (R1, R2), a non-wired Au electrode (R3) and bare ReS_2_ (R4). R1 and R2 dominate; the plotted value for R2 is the absolute magnitude because its sign is opposite by geometry. Both R3 and R4 sit near the noise floor, indicating minimal optical or electrical crosstalk across unaddressed electrodes and a short carrier diffusion length under zero bias (see Supporting Information Sections S3 and S4 for more details).

Figure 2e,g demonstrate the device’s dynamic characteristics and robustness at a wavelength of 650 nm. The transient under chopped illumination yields a characteristic time of ~25 ms (Figure 2e), consistent with an RC-limited photovoltaic junction rather than slow photogating. Under extended cycling, the photocurrent exhibits stable plateaus and reproducible on/off transitions with no discernible drift (Figure 2g). Together, these data establish that the star pixel operates in a genuine self-powered photovoltaic mode, with junction-confined generation, strong immunity to lateral crosstalk, and a millisecond-scale response suitable for imaging readout. In addition, ReS_2_ as a narrow-bandgap material, has been demonstrated to exhibit broadband photoresponse [11]. We further evaluated the dynamic photoresponse of the Au/ReS_2_ photodetector at 940 nm, confirming its potential for infrared operation (see Section S5 of the Supporting Information).

Figure 2 illustrates the zero-polarization photoresponse based on the PTE effect at the Au/ReS_2_ metal–semiconductor junction. Next, we examine how the same star pixel maps the linear polarization angle into an electrical signal, and how multi-junction readout increases amplitude without applied bias. All measurements here are taken under short-circuit conditions, with the focused spot positioned at the junction region identified previously. The in-plane polarization angle θ is rotated while the probed source–drain pair remains fixed. Crystal axes are registered by angle-resolved Raman (Figure 1e): the Re-chain (b-axis) is misaligned from the vertical electrode axis by ~3°, which we treat as a fixed phase offset in the fits.

**Figure 3:**
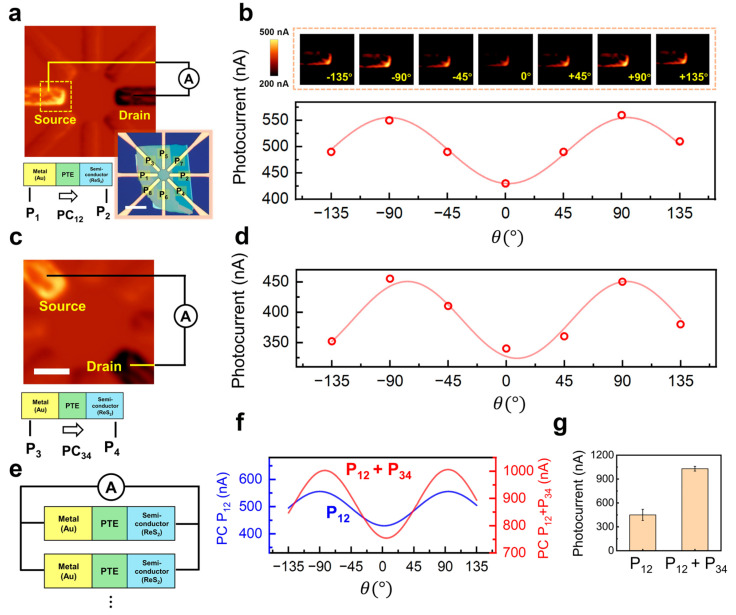
Linear polarization encoding and multi−junction electrical superposition. (**a**) Device schematic with two readout pairs, P_12_ and P_34_. (**b**) Short-circuit photocurrent of P_12_ versus polarization angle *θ* at zero bias; data fit $I\left(\theta \right)=I_{0}+I_{\alpha }cos\left(2\left(\theta -\varphi \right)\right)$ with φ consistent with the Raman-derived b−axis offset (~3°). (**c**,**d**) The nominally near-orthogonal pair P_34_ shows a complementary channel. Inset scale, 6 μm (**e**) Schematic of multi-junction electrical superposition. (**f**) Comparison of $I\left(\theta \right)$ for P_12_ alone and the summed output P_12_ + P_34_: superposition increases amplitude without altering phase or periodicity. (**g**) Angle-averaged currents: the summed readout approaches twice that of a single channel.

Figure 3a illustrates the short-circuit photocurrent of P_12_, which varies with *θ* in a canonical two-lobe, 180° periodic pattern (Figure 3b) that is well fit by

(4) $$\;\;I\left(\theta \right)=I_{0}+I_{\alpha }cos\left(2\left(\theta -\varphi \right)\right)\;$$

where $I_{0}$ is the mean level, $I_{\alpha }$ the modulation amplitude, and φ the fixed offset between the laboratory zero and the crystal b-axis. The fitted φ matches the Raman registration (~3°), indicating that maxima/minima occur when the incident field is parallel/orthogonal to the high-absorption axis of ReS_2_. Under zero bias, the built-in field at the illuminated junction dominates carrier separation, so the angular dependence primarily reflects anisotropic absorption (with a smaller contribution from anisotropic mobility) rather than transport bottlenecks. Modest deviations from a perfect cosine near the extrema can be attributed to local barrier nonuniformity and spot overlap at the metal edge; these do not affect the robust extraction of phase and contrast.

Having validated a single pair, we introduce a second pair P_34_ (Figure 3c) to obtain a complementary-phase channel. Its angular dependence likewise follows cos 2θ (Figure 3d), consistent with the geometric rotation of the electrode axis with respect to the crystal. The modulation depths are similar; minor amplitude differences likely arise from contact geometry, local coverage thickness, or other process variations. At this point, the two-channel description is complete: the phase φ recovers the polarization angle, and the modulation depth $I_{\alpha }/I_{0}$ quantifies sensitivity.

Building on these two channels, we exploit the star pixel’s parallel multi-junction nature to increase the absolute signal via electrical superposition without degrading angular selectivity (schematic in Figure 3e). In short-circuit mode, each junction exposed to light acts as an independent PTE power source. Figure 3f compares $I\left(\theta \right)$ from P_12_ alone to the summed output P_12_ + P_34_: the superposed trace shows a uniform amplitude increase across angle while preserving the sinusoidal dependence and phase set by the material anisotropy. The angle-averaged statistics in Figure 3g quantify this gain—two junctions of comparable strength add nearly linearly at short circuit, giving close to a two-fold increase in mean current. If noise in different junctions is approximately uncorrelated (as supported by Figure 2, which shows low inter-electrode crosstalk and highly localized response), the signal-to-noise ratio is expected to scale roughly as $\sqrt{N}$ with the number *N* of combined channels. The star geometry provides eight spokes at 45° spacing; one can therefore select phase-matched subsets to realize scalable gain without sacrificing modulation depth. Conversely, indiscriminate summation over a broad angular span may dilute contrast; our ‘phase-matching’ strategy avoids this, with practical limits set mainly by series resistance and the readout RC constant.

Table 1 compares our Au/ReS_2_ photodetector with several related devices. Benefiting from deliberate device design and careful control of the spot size and position, our device operates in a self-powered mode, enables a tunable polarization ratio without the need for a gate, and exhibits a reasonably fast response speed.

**Figure 4:**
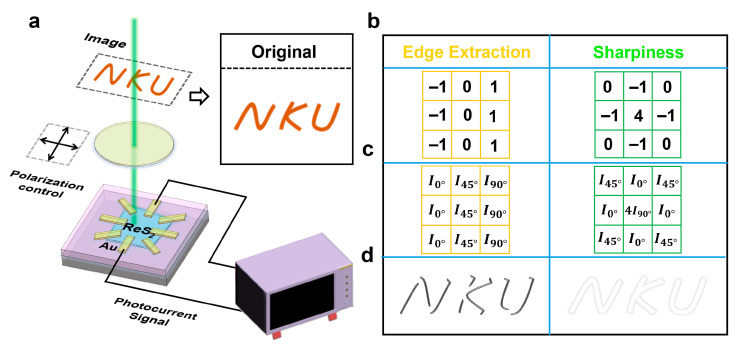
Polarization-resolved imaging and on-pixel polarization convolution. (**a**) Minimal imaging setup with a projected ‘NKU’ pattern, a rotatable linear polarizer, and zero-bias readout from the eight-terminal Au-ReS_2_ pixel. (**b**) Schematic of electronic weighting of polarization channels to emulate spatial operators. (**c**) Edge-extraction output obtained from $I_{90^{{}^{\circ}}}-I_{0^{{}^{\circ}}}$, highlighting stroke boundaries with minimal background. (**d**) Sharpness enhancement output suppressing low-frequency background and improving edge definition.

Having shown that each star pixel delivers independent, phase-registered polarization channels with millisecond dynamics (Figure 2 and Figure 3), we assembled a minimal imaging experiment to test scene reconstruction and on-pixel feature extraction (Figure 4a). A stencil (‘NKU’) is projected onto the device plane; a rotatable linear polarizer sets the incident polarization; and the Au-ReS_2_ star is read at zero bias. For each field point (single-pixel raster scan in this proof-of-concept), we record short-circuit currents for three representative analyzer angles, $\theta =\left\{0^{{}^{\circ}},45^{{}^{\circ}},90^{{}^{\circ}}\right\}$, referenced to the crystal b-axis determined in Figure 1. An ‘intensity-only’ image is obtained by equally weighting the three channels—effectively the per-pixel average, which serves as a baseline and confirms uniform illumination. The central idea is to treat the three polarization responses as a local basis and perform linear combinations to emulate spatial operators without additional optics or bias. Denoting the measured trio at a pixel by the vector $I={\left[I_{0^{{}^{\circ}}},I_{45^{{}^{\circ}}},I_{90^{{}^{\circ}}}\right]}^{T}$, we compute a processed output:

(5) $$Y=W\cdot I=\sum_{k}\omega_{k}I_{\theta_{k}}$$

with W chosen to realize a desired operator. This is analogous to a 3 × 3 convolution, but the ‘kernel’ acts in polarization space (the three analyzer orientations) rather than by mixing neighboring pixels. Two examples are shown in Figure 4b. The first one is the Edge extraction. Pairing the spatial kernel

(6) $$\;\;\;\left[\begin{array}{ccc} -1 & 0 & 1\\ -1 & 0 & 1\\ -1 & 0 & 1 \end{array}\right]\;$$

with the polarization channel matrix

(7) $$\;\;\left[\begin{array}{ccc} I_{0^{{}^{\circ}}} & I_{45^{{}^{\circ}}} & I_{90^{{}^{\circ}}}\\ I_{0^{{}^{\circ}}} & I_{45^{{}^{\circ}}} & I_{90^{{}^{\circ}}}\\ I_{0^{{}^{\circ}}} & I_{45^{{}^{\circ}}} & I_{90^{{}^{\circ}}} \end{array}\right]\;$$

reduces algebraically to

(8) $$\;\;E\propto I_{90^{{}^{\circ}}}-I_{0^{{}^{\circ}}}\;\;$$

a difference of orthogonal channels that suppresses polarization-insensitive background and highlights features that rotate the local E-field or preferentially transmit one orientation.

Another example is the Sharpness enhancement. Applying

(9) $$\;\;\;\left[\begin{array}{ccc} 0 & -1 & 0\\ -1 & 4 & -1\\ 0 & -1 & 0 \end{array}\right]\;$$

to

(10) $$\;\left[\begin{array}{ccc} I_{45^{{}^{\circ}}} & I_{0^{{}^{\circ}}} & I_{45^{{}^{\circ}}}\\ I_{0^{{}^{\circ}}} & 4I_{90^{{}^{\circ}}} & I_{0^{{}^{\circ}}}\\ I_{45^{{}^{\circ}}} & I_{0^{{}^{\circ}}} & I_{45^{{}^{\circ}}} \end{array}\right]$$

yields

(11) $$\;S\propto {4I}_{90^{{}^{\circ}}}-{4I}_{0^{{}^{\circ}}}\;\;$$

which behaves as a polarization-domain Laplacian, accentuating high-frequency content while rejecting slowly varying intensity. In practice, the constant scale factors only set overall gain and can be absorbed into readout.

Figure 4c,d show the weight–channel pairings and corresponding outputs from the stitched scan images. The edge operator yields sharp responses along letter strokes and corners with minimal haloing, consistent with the $I_{90^{{}^{\circ}}}-I_{0^{{}^{\circ}}}$ differential that removes uniform background. The sharpness operator further enhances stroke clarity while suppressing low-frequency noise. Since all operations use self-powered currents at the device plane, no mechanical analyzer switching, bias routing, or active modulation is required. The star-shaped geometry can incorporate additional analyzer angles for custom filters or Stokes-like reconstructions, and the millisecond response with low crosstalk (Figure 2) enables implementation either through direct current summation or digital post-processing. These results confirm that a single Au-ReS_2_ Schottky pixel can function as a polarization-aware computational sensor, supporting compact, scalable polarization imaging and on-chip pattern recognition.

## 3. Conclusions

In this study, a self-powered polarization-sensitive photodetector was developed based on an eight-terminal star-shaped Au/ReS_2_ metal–semiconductor junction array. Based on the PTE effect, the device operates in a self-powered mode, with the photoresponse localized at the Au/ReS_2_ interface, effectively eliminating dark current and bias-induced noise. Through precise crystallographic orientation, we exploited the inherent in-plane anisotropy of ReS_2_ to achieve the angle-resolved detection of linearly polarized light. By electrically superposing the outputs of the phase-matched channels, the signal amplitude was increased while maintaining the modulation depth, providing a method for directly modulating the polarization ratio and improving the signal-to-noise ratio without an external power supply. The concept has been verified by polarization-resolved imaging and pattern recognition using a minimum pixel unit, highlighting the potential of channel combination as a polarization domain function operator. These results construct a compact and scalable platform for polarization-resolved photodetection and pixel-level computational sensing.

## Figures and Tables

**Table 1:** Comparison of Au/ReS_2_ photodetectors with similar reported polarization devices.

Device Structure	Working Mechanism	Self-Powered	Polarization Ratio Modulation	Response Time	Refs.
Au/ReS_2_	Photoconductive	No	No	-	[2]
Au/ReS_2_	Photoconductive	No	Gate voltage	2.6 ms	[11]
Au/ReS_2_ (suspended)	Photoconductive	No	No	83.5 μs	[15]
ReSe_2_/WSe_2_	Photovoltaic	Yes	Gate voltage	197 μs	[31]
Perovskite	Photovoltaic	Yes	Ferro-pyro-phototronic effect	-	[32]
Au/ReS_2_	PTE	Yes	Parallel multi-junction	25 ms	This work

## Data Availability

All of the data that support the findings of this study are reported in the main text and Supplemental Document. Source data are available from the corresponding author upon reasonable request. Correspondence and requests for materials should be addressed to Z.B. Liu.

## Supplementary Materials

The following supporting information can be downloaded at https://www.mdpi.com/article/10.3390/s25175375/s1, Figure S1. Schematic illustration of fabrication processing; Figure S2. Wide range *I-V* characteristic curve of Au/ReS_2_ photodetector. To confirm whether the I-V curve would bend at higher bias voltages, we tested a wide range of I-V curves; Figure S3. Schematic illustration of the Shockley–Ramo interpretation for photocurrent mapping in an eight-terminal radial device; Figure S4. Long-term cycling with stable on/off plateaus. Test wavelength, 940 nm. Figure S4 presents the photoelectric response of the device under 940 nm near-infrared illumination. When subjected to an incident power comparable to that at 633 nm (approximately 15 mW), the generated photocurrent reaches about 15 nA. Notably, even after extended cycling, the response maintains a stable plateau with reproducible on/off switching and shows no observable drift. Reference [33] is cited in Supplementary Materials.
