# Supplementary material for: Self-Powered Au/ReS2 Polarization Photodetector with Multi-Channel Summation and Polarization-Domain Convolutional Processing
Source: Sensors (Basel). 2025 Sep 1;25(17):5375. doi: 10.3390/s25175375 (PMC12431534; doi:10.3390/s25175375)
Supplement: Supplementary file 1 [file sensors-25-05375-s001.zip › sensors-3844533-supplementary.pdf]

Supporting Information for

Self-Powered Au/ReS_2 Polarization Photodetector with Multi-Channel Summation and Polarization-Domain Convolutional Processing

Ruoxuan Sun [1,†], Guowei Li [1,†], and Zhibo Liu [1,2,*]

[1] *The Key Laboratory of Weak Light Nonlinear Photonics, Ministry of Education, School of Physics and Teda Applied Physics Institute, State Key Laboratory of Photovoltaic Materials and Cells, Nankai University, Tianjin 300071, China*

[2] *The collaborative Innovation Center of Extreme Optics, Shanxi University, Taiyuan, Shanxi 030006, China*

∗ *Corresponding author: liuzb@nankai.edu.cn*

Section S1. Fabrication of ReS_2-Based Optoelectronic Devices.

ReS_2 crystals were mechanically exfoliated from bulk using 3M Scotch tape and deposited onto a Si substrate with a thermally grown oxide layer. Candidate flakes with suitable thickness and lateral dimensions were identified using an optical microscope. To transfer the selected flake, a PDMS film was gently placed over the sample, and a small amount of deionized water was introduced between the flake and the Si substrate. This water-assisted method reduced adhesion to the original substrate, enabling the ReS_2 flake to adhere to the PDMS surface. The PDMS stamp carrying the flake was then aligned to a clean target substrate under a microscope, and mild heating was applied to promote adhesion and release the ReS_2 onto the new substrate without introducing significant strain or contamination.

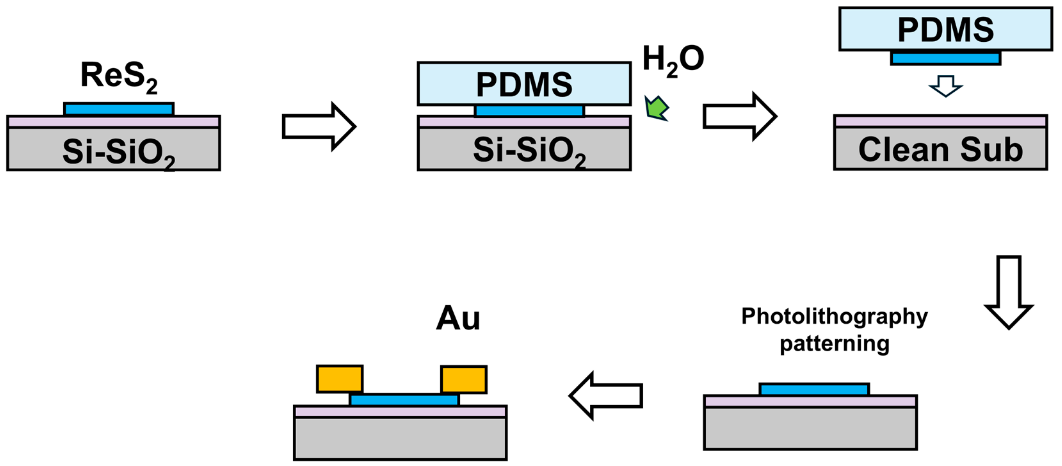

Figure S1. Schematic illustration of fabrication processing.

After transfer, photolithography was employed to define the electrode regions. The process was carried out under a nitrogen atmosphere to minimize oxidation and contamination of the ReS_2 surface. During exposure, the laser power was carefully reduced to prevent thermal or photo-induced damage to the material. The pH of the developer solution was lowered to suppress chemical etching of the flake edges. Subsequently, Au electrodes were deposited via magnetron sputtering, with the sputtering power finely tuned to reduce energetic ion bombardment and mitigate Fermi level pinning at the Au–ReS_2 interface. These precautions ensured the preservation of the intrinsic optoelectronic properties of the ReS_2 channel and improved the stability and reproducibility of the final device.

Section S2. Wide range *I-V* characteristic curve of Au/ReS2 photodetector..

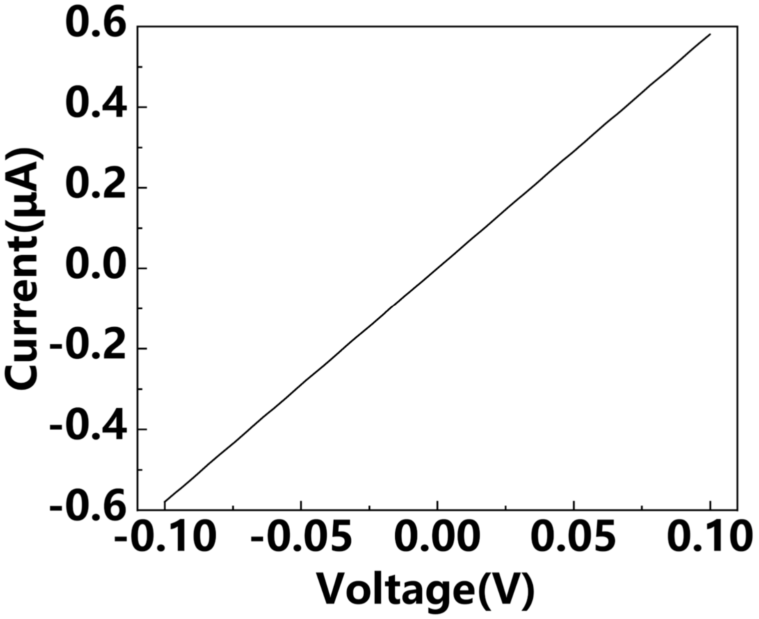

Figure S2. Wide range *I-V* characteristic curve of Au/ReS_2 photodetector.

To confirm whether the I-V curve would bend at higher bias voltages, we tested a wide range of I-V curves.

Section S3. High-Precision Scanning Photocurrent Imaging System.

To precisely characterize the spatially resolved photo-response of optoelectronic devices, a high-precision scanning photocurrent imaging system was developed. Built upon the WiTec Alpha300 RAS confocal Raman microscopy platform and integrated with an external source meter and a high-accuracy piezoelectric translation stage, this setup enables point-by-point photocurrent measurements on two-dimensional material heterostructures. During measurements, a 532 nm continuous-wave laser is focused through a 50× long working distance objective to form a near-Gaussian spot of approximately 500 nm in diameter. The device, mounted on the piezoelectric scanning stage, is translated in the X–Y plane to raster-scan the entire active area. To minimize drift and maintain stable focusing, all optical components remain fixed while only the sample is moved, ensuring consistent measurement conditions.

Photocurrent signals are acquired using either a Keithley 2634B dual-channel source meter or the source meter module integrated into the WiTec system. The measured

currents are mapped to their corresponding spatial coordinates to produce two-dimensional photocurrent images, revealing local variations in photo-response and carrier transport behavior. Experimental parameters such as current range, integration time, and scan speed are optimized according to the device properties to achieve a balance between signal fidelity and dynamic response. Post-processing, including background subtraction and image smoothing, is applied to enhance image contrast and suppress noise, thereby providing high-quality photocurrent maps for further analysis.

Section S4. Shockley–Ramo Interpretation of Eight-Terminal Photocurrent Measurements.

In this experiment, scanning photocurrent imaging was performed on a gapless material using an eight-terminal radial structure. When a pair of opposite electrodes on the left and right served as the current-collecting terminals, the photocurrent signals near these electrodes were significantly stronger than those near the other six floating electrodes. This phenomenon can be consistently explained within the Shockley–Ramo framework, in which the photocurrent is expressed as [1]:

$$I \propto J_{ph(r)} \cdot \nabla\psi(r) d^2 r \qquad \text{(S1)}$$

where $J_{ph(r)}$ is the local photocurrent and $\nabla\psi(r)$ is the weighting field determined by the device geometry and boundary conditions at the contacts. The boundary conditions imposed by the connected electrodes concentrate the $\nabla\psi(r)$ field lines primarily between them, thereby assigning the largest integration weight to the photocurrent generated in these regions and resulting in a markedly enhanced signal amplitude.

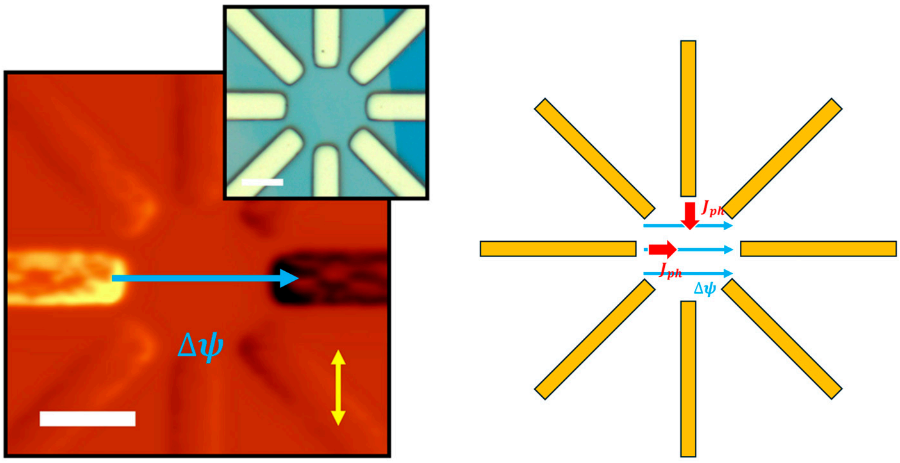

Figure S3. Schematic illustration of the Shockley–Ramo interpretation for photocurrent mapping in an eight-terminal radial device.

In addition, the photocurrent amplitude exhibits pronounced directionality. Since only the component of the photogenerated current parallel to the weighting field couples effectively, the connected left and right electrodes, which establish $\nabla\psi(r)$ predominantly along the horizontal direction, yield the strongest response from interfaces or local inhomogeneities aligned with this field. In contrast, the orientations of the other six radial floating electrodes form finite angles with $\nabla\psi(r)$, resulting in smaller or even sign-reversed $J_{ph(r)} \cdot \nabla\psi(r)$ projections and, consequently, a much weaker net signal.

For floating electrodes, the weighting field at the electrode surface exhibits a sign-reversing dipolar distribution, with the side facing the current-collecting pair and the opposite side producing normal components of opposite sign. This leads to partial cancellation of the local photocurrent. The combined effects of geometry and boundary conditions give rise to two salient features of the global photocurrent response: (i) globality, where the signal magnitude is governed primarily by the weighting field distribution rather than by the distance between the excitation spot and the contacts; and (ii) directionality, where the signal strength and polarity depend on the angle between the photocurrent and the weighting field. These characteristics are in excellent agreement with the experimental observations and provide a clear physical picture for understanding complex photocurrent patterns in gapless materials.

Section S5. Time-dependent photoresponse at 940 nm wavelength.

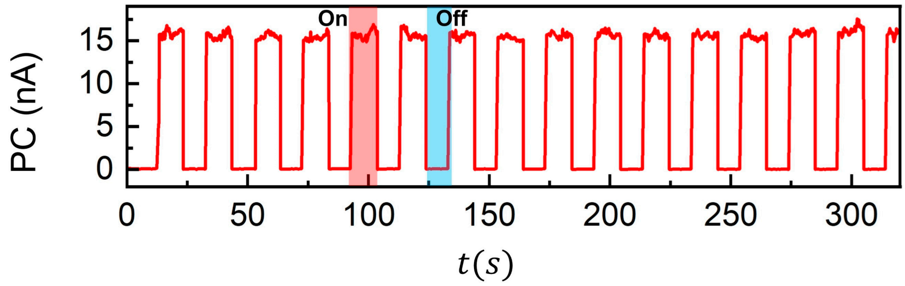

Figure S4. Long-term cycling with stable on/off plateaus. Test wavelength, 940 nm.

Figure S4 presents the photoelectric response of the device under 940 nm near-infrared illumination. When subjected to an incident power comparable to that at 633 nm (approximately 15 mW), the generated photocurrent reaches about 15 nA. Notably, even after extended cycling, the response maintains a stable plateau with reproducible on/off switching and shows no observable drift.

References

1. Song, J. C.; Levitov, L. S., Shockley-Ramo theorem and long-range photocurrent response in gapless materials. Physical Review B 2014, 90, (7), 075415.
